# ﻿Two new *Galendromimus* species (Acari, Phytoseiidae) from Brazil, with a world key and distributional notes on the tribe Galendromimini

**DOI:** 10.3897/zookeys.1260.170115

**Published:** 2025-11-20

**Authors:** Amanda Conceição dos Santos, Peterson Rodrigo Demite, Felipe Micali Nuvoloni, Antonio Carlos Lofego

**Affiliations:** 1 Department of Biological Sciences, Graduate Program in Biodiversity, São Paulo State University (UNESP), São José do Rio Preto, São Paulo, Brazil São Paulo State University São Paulo Brazil; 2 Campus Bonfim, Instituto Federal de Roraima (IFRR), Bonfim, Roraima, Brazil Instituto Federal de Roraima Bonfim Brazil; 3 Centro de Formação em Ciências Ambientais, Universidade Federal do Sul da Bahia (UFSB), Porto Seguro, Bahia, Brazil Universidade Federal do Sul da Bahia Porto Seguro Brazil

**Keywords:** Biodiversity, biogeographic, morphology, predatory, systematics, taxonomy

## Abstract

Two new species of *Galendromimus* Muma are described based on female and male specimens collected from native plants in the Atlantic Forest of Bahia State, northeastern Brazil: Galendromimus (Galendromimus) primulaporis Santos, Demite & Lofego, **sp. nov.** and Galendromimus (Galendromimus) striatusornatus Santos, Demite & Lofego, **sp. nov.** The tribe Galendromimini comprises predatory mites of ecological and agricultural importance. Although well studied in some regions, the diversity and distribution of Phytoseiidae in tropical areas remain incompletely known. Besides the species descriptions, we provide a global distribution summary and an updated identification key for all known Galendromimini species. These contributions improve taxonomic resolution and biogeographic knowledge, facilitating future ecological, evolutionary, and applied research.

## ﻿Introduction

Phytoseiidae (Acari: Mesostigmata) comprises more than 2,700 valid species worldwide (Demite et al. 2025) that are considered to play an important role in terrestrial ecosystems as natural enemies of phytophagous mites and small insects (McMurtry and Croft 1997; Gerson et al. 2003). Due to their ecological relevance and economic importance in biological control programmes, phytoseiids have been the focus of extensive faunistic and taxonomic studies in Brazil (Demite et al. 2014a; Lofego et al. 2024). However, significant gaps remain in our knowledge of their diversity and distribution in tropical and subtropical regions, where high species richness suggests that much remains undocumented.

The tribe Galendromimini (Typhlodrominae) comprises 18 species distributed across four genera: *Galendromimus* Muma, *Cydnoseius* Muma, *Silvaseius* Chant & McMurtry and *Breviseius* Moraes, Barbosa & Castro (Demite et al. 2025). Species of *Galendromimus* are characterised by the absence of setae *S2*, *S4*, and *R1*, and the presence of setae *s6* and *Z1*, with further variation in the presence or absence of setae *z3*, *J2*, *S5*, *JV3*, *JV4*, and *ZV3* (Chant and McMurtry 1994; Demite et al. 2014b; Silva et al. 2024).

Two subgenera are currently recognised within *Galendromimus*: G. (Galendromimus) and G. (Nothoseius) (Chant and McMurtry 1994). The subgenera of *Galendromimus* are primarily separated by idiosomal setae: G. (G.) with *J2* and *JV3* present, *JV4* and *ZV3* absent; G. (N.) with *J2* and *JV3* absent, *JV4* and *ZV3* present. The subgenus G. (Galendromimus) comprises seven species: G. (G.) alveolaris (De Leon, 1957), G. (G.) sanctus De Leon, 1967, G. (G.) tunapunensis De Leon, 1967, G. (G.) paulista Zacarias & Moraes, 2001a, G. (G.) multipoculi Zacarias, Moraes & McMurtry, 2002, G. (G.) roraimensis Demite & Lofego, 2014, and G. (G.) kynolithus Silva, Gondim Jr & Demite, 2024. Most species in this subgenus are restricted to South America and Trinidad, except for G. (G.) alveolaris, which has been reported throughout the America continent (Demite et al. 2025).

The presence or absence of *z3* and *S5* is used to divide the subgenus Galendromimus (Galendromimus) into three species groups: the *sanctus* group (both setae absent), the *roraimensis* group (*z3* present and *S5* absent), and the *alveolaris* group (*S5* present and *z3* absent) (Chant and McMurtry 1994; Demite et al. 2014b; Silva et al. 2024). Currently, the *alveolaris* group comprises four species: G. (G.) alveolaris, G. (G.) paulista, G. (G.) multipoculi, and G. (G.) tunapunensis.

In this study, we describe two new species of G. (Galendromimus) based on both female and male specimens, present an updated identification key for all known Galendromimini species, and provide a summary of their global distribution.

## ﻿Materials and methods

Specimens of *Galendromimus* were collected from leaf samples of the following plant species *Henriettea succosa* (Aubl.) DC. (Melastomataceae), *Vismia atlantica* L. Marinho & M.V. Martins (Hypericaceae) and Inga vera Willd. subsp. affinis (DC.) T.D. Penn (Fabaceae). These plants belong to the natural vegetation of two National Conservation Units located within the Atlantic Rainforest biome: Parque Estadual da Serra do Conduru (14°29'52.6"S, 39°08'45.3"W), and Reserva Ecológica Michelin (13°49'17.2"S, 39°11'55.1"W) Bahia State, Brazil.

The mites were mounted on slides using Hoyer’s medium and examined under a phase-contrast microscope (Zeiss AX10). Images were captured with an attached camera (V6 38MP FHD HDMI). Illustrations of the species were prepared using Sketchbook application (desktop version) and Adobe Illustrator ®.

Measurements of morphological structures were taken using a graduated eyepiece and are presented in micrometres (μm) in the text. Measurements of the holotypes are presented in bold, followed by the average, with minimum and maximum values in parentheses for the holotype and paratypes, when available. The setal nomenclature follows that of Lindquist and Evans (1965), adapted by Rowell et al. (1978) and Lindquist (1994) for the dorsum. The setal nomenclature for the venter is as proposed by Chant and Yoshida-Shaul (1991), and the idiosomal setal pattern follows Chant and Yoshida-Shaul (1992).

Type specimens are deposited in the Acari Collection of Departamento de Ciências Biológicas, Universidade Estadual Paulista (**UNESP**), São José do Rio Preto, State of São Paulo, Brazil (DZSJRP - Acari).

To assess the global distribution of Galendromimini species by country, we retrieved data from the Phytoseiidae Database (Demite et al. 2025). Only valid species were included. Additionally, the identification key for species of the tribe Galendromimini was updated from Silva et al. (2024) by incorporating the two new species described in this study.

## ﻿Results and discussion

### ﻿ Galendromimus

Taxon classificationAnimaliaMesostigmataPhytoseiidae

Muma, 1961

1C19E57A-46D0-5592-AB5B-B939745ABC6F

#### Note.

The genus is characterised by the presence of setae *s6* and *Z1* and the absence of *S2*, *S4*, and *R1*; setae *z3*, *J2*, *S5*, *JV3*, *JV4*, and *ZV3* may be present or absent. Some setae on the dorsal shield, especially *Z4* and *Z5*, are elongate, thick, and strongly serrated (Muma 1961; Chant and McMurtry 1994; Demite et al. 2014b).

### ﻿ Galendromimus (Galendromimus)

Taxon classificationAnimaliaMesostigmataPhytoseiidae

Chant & McMurtry, 1994

DC3E3B24-EF24-5B2C-B9E2-8FBB5A9B7BD1

#### Note.

Characterised by the presence of *J2* and *JV3* and the absence of *JV4* and *ZV3*; chelicerae with few teeth and spermatheca cervix elongate tubular (Chant and McMurtry 1994).

### ﻿ Galendromimus (Galendromimus) primulaporis

Taxon classificationAnimaliaMesostigmataPhytoseiidae

Santos, Demite & Lofego sp. nov.

F452144A-2169-5BEA-B4A2-B730E5BF958E

https://zoobank.org/39FD3C85-CA4F-462F-B278-0915DF14F303

Figs 1, 2, 3

#### Type specimens.

***Holotype***: Brazil; 1 ♀; Parque Estadual da Serra do Conduru, Uruçuca, Bahia; 14°29'52.6"S, 39°08'45.3"W; 5 Jul. 2023; A.C. Santos leg.; on *Henriettea succosa* (Aubl.) DC. ***Paratypes***: 2 ♀♀, same data as holotype; 2 ♀♀ and 2 ♂♂, same data as holotype, but on *Vismia atlantica* L. Marinho & M.V. Martins; 2 ♂♂, same data as holotype, but from Inga vera Willd. subsp. affinis (DC.) T.D.Penn.

#### Diagnosis.

Idiosomal setal pattern (11D:6B; JV-4: ZV-3). Dorsal shield reticulated, with a pattern of reticulation surrounding each pore (*gd2*, *gd4*, *gd5*, *gd6*, *gd8*, and *gd9*) constitutes reminiscent of a daisy flower; setae *z2*, *z4*, z5, *Z1*, *s4*, *s6*, *S5*, *j1*, *j3*, *j4*, *j6*, and *J2* smooth; setae *Z4* and *Z5* serrated and distinctly thick. Only the tubercles of setae *Z4* and *Z5* large and distinctly visible; seta *r3* located in lateral integument next to dorsal shield margin; seta *R1* absent; peritreme extending to the level of seta *z2*; ventrianal shield longer than wide, with a distinct waist at *JV2* level, smooth anterior to anal opening and with reticulations laterally and posteriorly, with four pairs of preanal setae (*JV1*, *JV2*, *JV3*, and *ZV2*) and a pair of pores (*gv3*), located posterior to *JV2*; *JV4* and *ZV3* absent. Calyx of the spermatheca long and slender throughout, but bulbous near nodular atrium. Males with *JV1*, *JV2*, *JV3*, and *ZV2*; ventrianal shield smooth and subtriangular with slight reticulation on posterolateral margins to anal opening; spermatodactyl is slender and elongated. Trochanter I 1 0/1 0/2; genu II 2 2/1 2/0 0.

**Figure 1.**
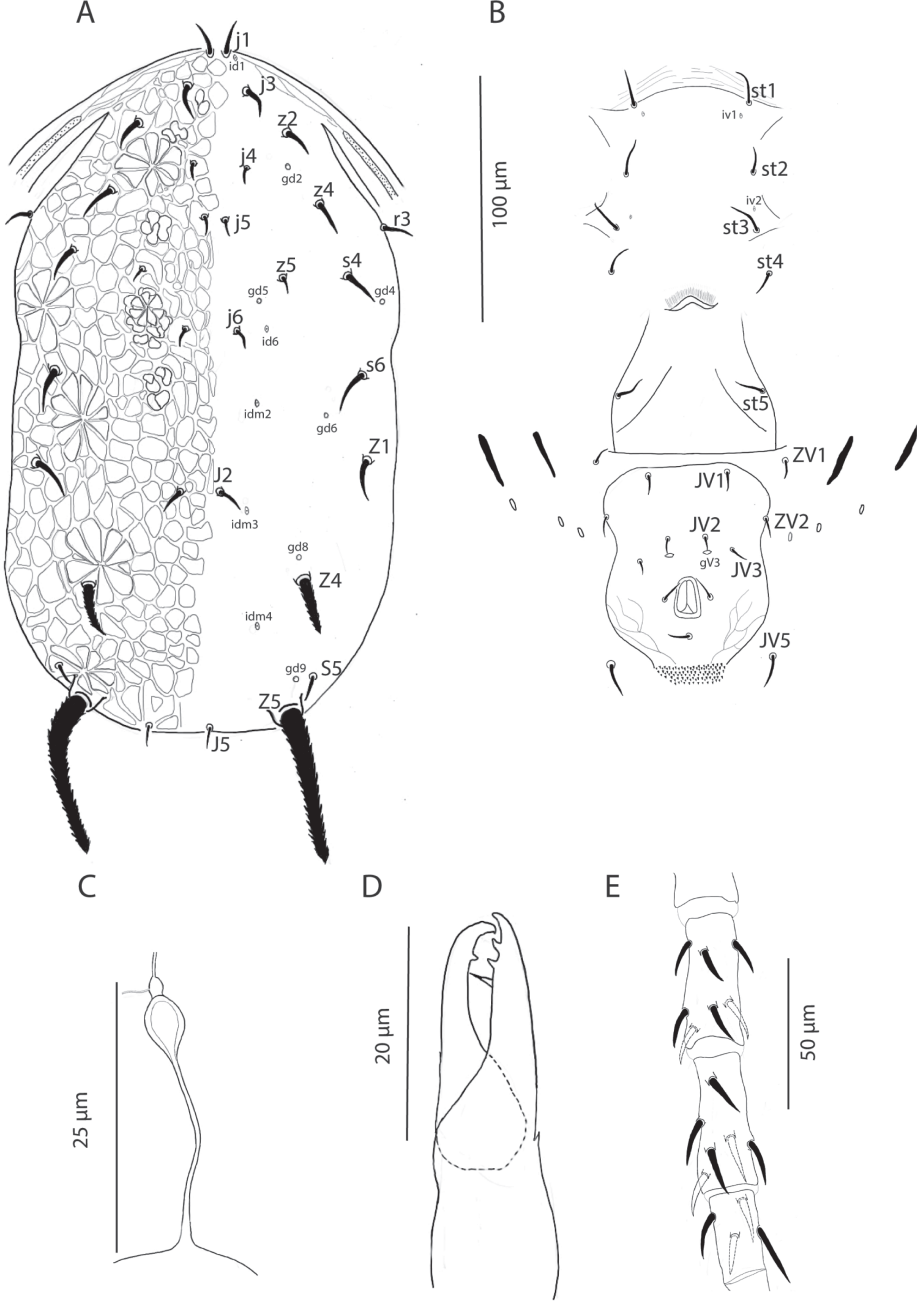
Galendromimus (Galendromimus) primulaporis sp. nov., female. **A.** Dorsal idiosoma; **B.** Ventral idiosoma; **C.** Spermatheca; **D.** Chelicera; **E.** Genu, tibia, and basitarsus of the leg IV.

Female (*n* = 5). ***Dorsum of idiosoma*** (Figs 1A, 3). Dorsal shield reticulate **270** 272 (266–279) long and **151** 152 (148–164) wide at the level of *s4*; six pairs of pores (solenostomes) (*gd2*, *gd4*, *gd5*, *gd6*, *gd8*, and *gd9*) all surrounded by a daisy-shaped reticulation pattern, and five pairs of lyrifissures (poroids) (*id1*, *id6*, *idm2*, *idm3*, and *idm4*) visible. Lengths of setae: *j1***15** 15 (14–15), *j3***14** 15 (14–16), *j4***8** 9 (8–10), *j5***9** 10 (9–10), *j6***10** 11 (10–13), *J2***14** 14 (13–15), *J5***12** 11 (11–12), *z2***15** 14 (12–16), *z4***16** 16 (16–17), *z5***8** 9 (8–11), *Z1***19** 20 (19–20), *Z4***26** 25 (25–27), *Z5***70** 71 (70–73), *s4***15** 16 (12–17), *s6***17** 20 (17–22), *S5***14** 14 (13–15) *r3***14** 14 (12–15). Seta *r3* located near the margin of the dorsal shield but its position may vary among individuals; in some specimens, the seta is situated at the margin of the dorsal shield. Only the tubercles of setae *Z4* and *Z5* large and distinctly visible. Setae *Z5* and *Z4* serrated and thick, all remaining setae smooth and acicular.

***Peritreme*** extending to approximately *z2* level.

***Venter*** (Fig. 1B). Sternal shield not completely discernible; distance between *st1*-*st3***53** 54 (53–55), *st1-st1***46** 46 (44–48), *st2-st2***51** 50 (50–52) and between *st3-st3***56** 56 (55–58). Metasternal plates not visible. Genital shield smooth, with distance between *st5-st5***60** 60 (54–64). Ventrianal shield longer than wide, **89** 91 (89–96) long, **64** 66 (64–66) wide at the level of *ZV2*; smooth anterior to anal opening and with scant posterolateral reticulation; with four pairs of preanal setae (*JV1*, *JV2*, *JV3*, and *ZV2*) and a small pair of rounded pores *gv3*, posteriad to *JV2*. Unsclerotised opisthogastric cuticle with two pairs of setae (*ZV1* and *JV5*). Two pairs of metapodal plates of similar size are present. All ventral setae smooth.

***Spermatheca*** (Fig. 1C). Calyx of the spermatheca long, slender throughout, but bulbous near nodular atrium; **25** 23 (22–26) long.

***Chelicera*** (Fig. 1D). Movable digit **22** 22 (19–24) long, with one subapical tooth, in addition to the apical tooth.; fixed digit **24** 21 (19– 22) long, with two teeth between pilus dentilis and apical tooth.

***Legs*** (Fig. 1E). In all legs, dorsal setae in genu, tibia and basitarsus are inserted in tubercles, whereas ventral setae lack tubercles. No macrosetae. Chaetotaxy as follows: trochanter I 1 0/1 0/2 1, genu I 2 2/1 2/1 2, genu II 2 2/1 2/0 0, genu III 1 2/1 2/0 1, genu IV 1 2/1 2/0 1.

**Figure 2.**
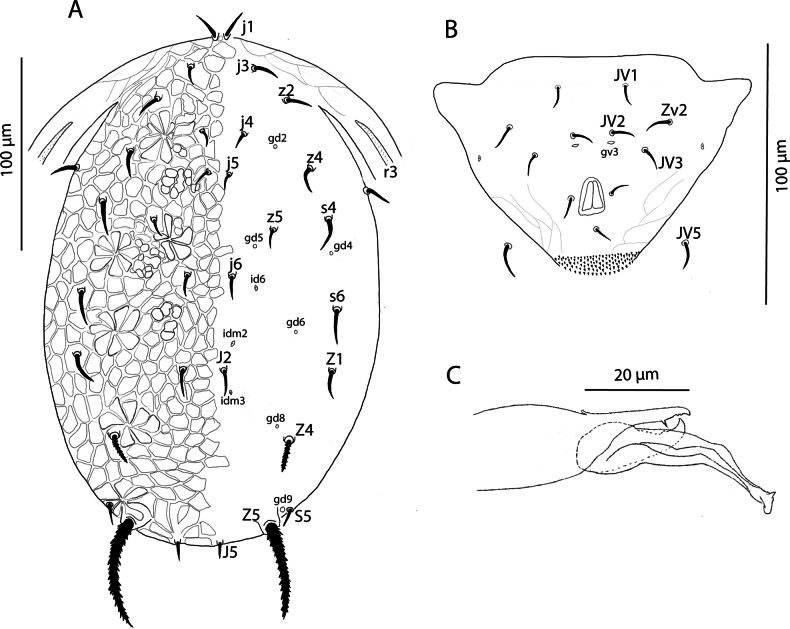
Galendromimus (Galendromimus) primulaporis sp. nov., male. **A.** Dorsal idiosoma; **B.** Ventrianal shield; **C.** Spermatodactyl.

Male (*n* = 4). ***Dorsum of idiosoma*** (Fig. 2A). Setal pattern and ornamentation of dorsal shield as in female; 215 (208–223) long and 132 (122–138) wide at *s4* level. With pairs of pores (*gd2*, *gd4*, *gd5*, *gd6*, *gd8*, and *gd9*), all surrounded by a daisy-shaped reticles set as in female, and only three pairs of lyrifissures (*id6*, *idm2*, and *idm3*) visible. Lengths of setae: *j1* 12 (10–14), *j3* 12 (11–13), *j4* 8 (6–9), *j5* 9 (8–10), *j6* 11 (8–13), *J2* 12 (11–15), *J5* 10 (9–11), *z2* 12 (11–13), *z4* 13 (10–15), *z5* 9 (8–11), *Z1* 15 (14–16), *Z4* 16 (15–18), *Z5* 47 (46–50), *s4* 15 (14–16), *s6* 17 (14–19), *S5* 10 (9–11), *r3* 12 (11–13). Seta *r3* located at the margin of the dorsal shield. Setae *Z4* e *Z5* serrated; setae *j1*, *j3*, *j4*, *j5*, *j6*, *J2*, *J5*, *r3*, *z2*, *z4*, *s4*, *s6*, and *S5* smooth; Only the tubercles of setae *Z4* and *Z5* large and distinctly visible. Setae *Z4* and *Z5* distinctly thick.

***Peritreme*** extending to region between the bases of *z4* and *z2*.

***Venter*** (Fig. 2B). Sternogenital shield smooth; ventrianal shield subtriangular and mostly smooth, except for slight reticulation posterolateral to anal opening; with a pair of lyrifissures close to the margin, at the level of *JV3*; 80 (75–85) long and 113 (110–119) wide at level of anterior corners, with four pairs of preanal setae (*JV1*, *JV2*, *JV3*, and *ZV2*). All ventral setae smooth. One distinct pair of pores (*gv3*) posteromesad *JV2*. *JV5* 10 (9–12) long.

***Chelicera*** fixed digit 19 (18–20) long with two apical teeth, movable digit 17 (16–18) long with one tooth.

***Spermatodactyl*** (Fig. 2C) is slender and elongated, curved distally, with a pointed, slightly expanded tip. Shaft 10 (9–11), foot 7 (5–8).

***Legs*** as in females.

#### Differential diagnosis.

Among the species of the *alveolaris* species group, females of the new species most closely resemble G. (G.) alveolaris, mainly in the shape of the spermatheca and the similar reticulation patterns arrangement of reticles surrounding the pores — although this latter pattern was not emphasised in the original description of G. (G.) alveolaris. Despite these similarities, G. (G.) alveolaris differs from the new species primarily by having the peritreme extending only to the level of *s4*, whereas in the new species it extends to the level of *z2*. It also differs in the length of seta *Z4* (61 µm), reaching the base of *Z5*.

Females of the remaining species of the group differ from the new species mainly as follows: G. (G.) paulista without *J2*; G. (G.) tunapunensis peritreme extending to level of *j1*; with *z4*, *Z1*, *Z4*, *s4*, and *s6* respectively ~ 2.1, 3.2, 2.7, 2.4 and 1.2× as long as in the new species; setae *z4*, *Z1*, *s4*, and *s6* serrated and thick; G. (G.) multipoculi with distinct pits in the central region of the dorsal shield and with *Z1*, *Z4*, *s4*, and *s6* respectively ~1.9, 1.7, 1.9, and 2.0× longer; G. (G.) striatusornatus sp. nov. has the spermathecal calyx broader near the atrium, but not forming a bulb as in G. (G.) primulaporis sp. nov. It also differs by having the peritreme extending to the level of seta *j1*, and setae *Z4* and *S5* are 1.8 and 1.6× longer, respectively, than in G. (G.) primulaporis sp. nov.

#### Remarks.

Plant parts (leaves and stems) of the host plants have various degrees of pubescence containing glandular and stellate trichomes. Such features may provide favourable microhabitats for mite occurrence, possibly offering shelter (Schmidt 2014).

#### Etymology.

**Figure 3.**
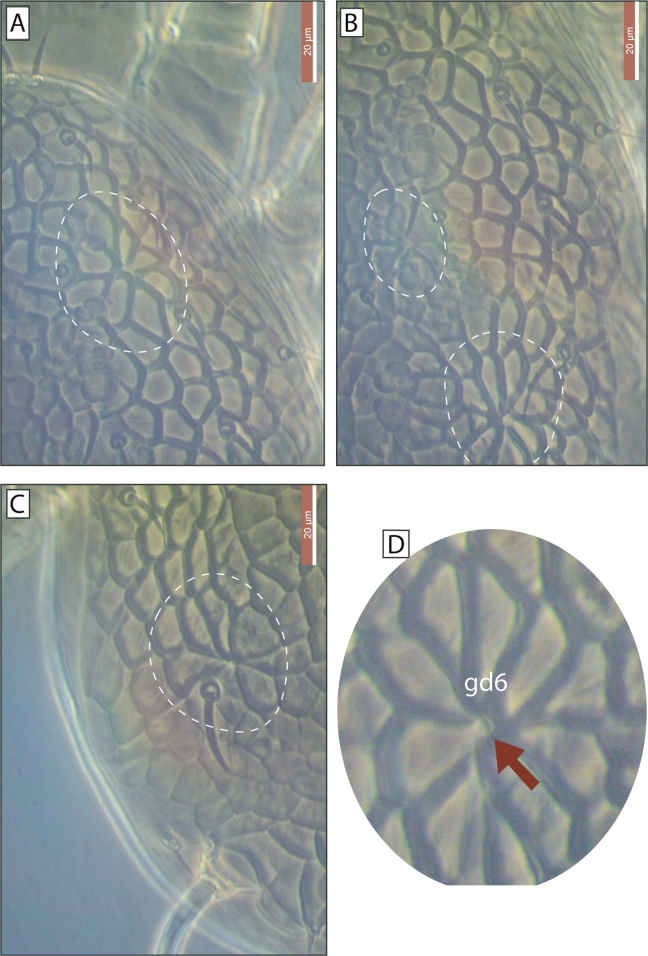
Ornamentation pattern surrounding the dorsal shield pores of Galendromimus (Galendromimus) primulaporis sp. nov. **A.***gd2*; **B.***gd5* and *gd6*; **C.***gd8*; **D** Enlarged *gd6* pore, highlighting the ornamentation pattern resembling daisies. Scale bars: 20 μm.

The name *primulaporis* is derived from the Latin *primula* (daisy) and *poris* (pores). It refers to the distinctive ornamentation pattern surrounding the dorsal shield pores, which resembles a form of daisy flowers (Fig. 3).

### ﻿ Galendromimus (Galendromimus) striatusornatus

Taxon classificationAnimaliaMesostigmataPhytoseiidae

Santos, Demite & Lofego sp. nov.

9D18E01B-5515-5081-AFCD-EE1DFD5A34EC

https://zoobank.org/A2C0464C-3EAA-48A0-9C7E-15F0D6DC3513

Figs 4, 5, 6

#### Type specimens.

***Holotype***: Brazil; 1 ♀; Reserva Ecológica Michelin, Igrapiúna, Bahia; 13°49'17.2"S, 39°11'55.1"W; 3 Jul. 2023; A.C. Santos leg.; on *Henriettea succosa* (Aubl.) DC. ***Paratype***: 2 ♂♂, same data as holotype.

#### Diagnosis.

Idiosomal setal pattern (11D:6B; JV-4: ZV-3). Dorsal shield ornamentation between setae *Z4* and *Z5* characterised by each reticle including a series of parallel ridges perpendicular to the longest margin; setae *z2*, *z4*, z5, *Z1*, *s4*, *j1*, *j3*, *j4*, *j6*, and *J2* smooth; setae *s6* and *J5* slightly serrate; Setae *Z4* and *Z5* strongly serrated and distinctly thick. All setae inserted on tubercles, but only the tubercles of setae *Z4* and *Z5* are large and distinctly visible; seta *r3* located at the margin of dorsal shield. Peritreme extending to the level of seta *j1*; posterior margin of sternal shield and metasternal plates not visible due to weak sclerotisation; genital shield smooth; ventrianal shield longer than wide, with reticulations laterally and posteriorly to the anal opening, with four pairs of preanal setae (*JV1*, *JV2*, *JV3*, and *ZV2*) and a pair of pores *gv3*, located posterior to *JV2*; *JV4* and *ZV3* absent; all ventral setae smooth. Calyx of the spermatheca with half next to vesicle tubular and the other half medially bulged, narrowing down at both extremes. Males with *JV1*, *JV2*, *JV3*, and *ZV2*, ventrianal shield subtriangular with reticulation on lateral margins; spermatodactyl C-shaped. Trochanter I 0 0/1 0/1 0 and genu II 2 2/0 2/0 1.

**Figure 4.**
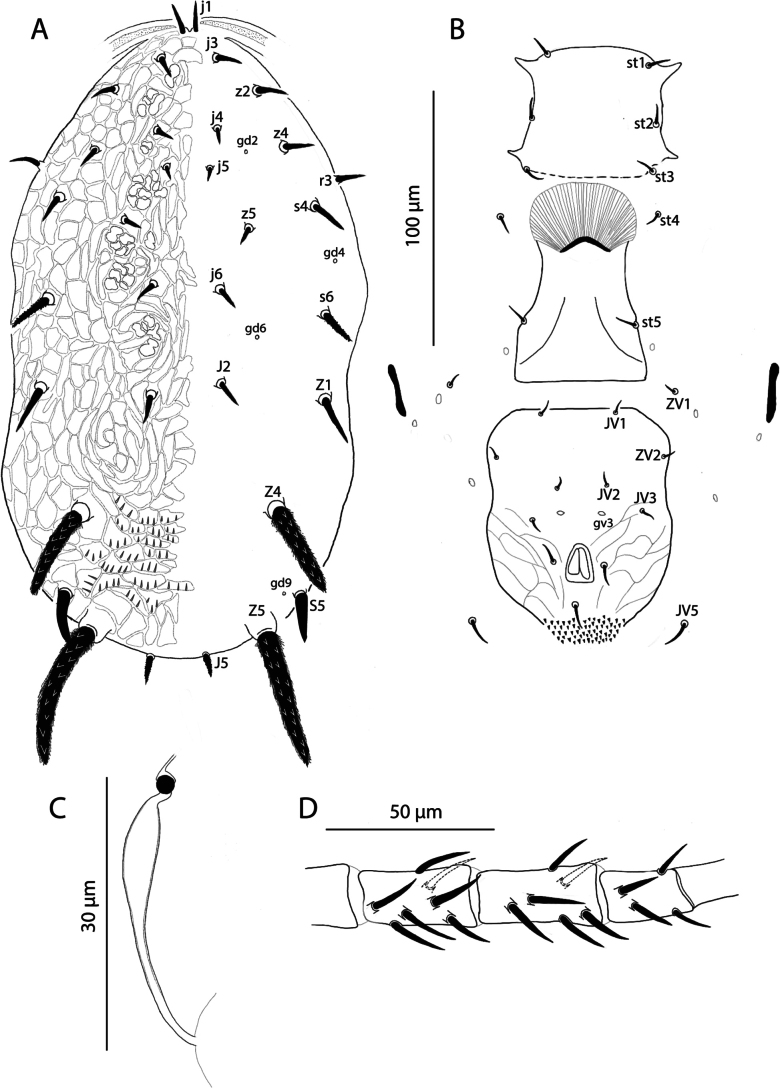
Galendromimus (Galendromimus) striatusornatus sp. nov., female. **A.** Dorsal idiosoma; **B.** Ventral idiosoma; **C.** Spermatheca; **D.** Genu, tibia and basitarsus of the leg IV.

Female (*n* = 1). ***Dorsum of idiosoma*** (Figs 4A, 6). Dorsal shield reticulate, **284** long and **151** wide at the level of seta *s4* with the ornamentation between setae *Z4* and *Z5* characterised by each reticle including a series of parallel ridges perpendicular to the longest margin; four pairs of pores (*gd2*, *gd4*, *gd6*, and *gd9*) visible; no lyrifissures visible. Lengths of setae: *j1***14**, *j3***13**, *j4***11**, *j5***14**, *j6***16**, *J2***20**, *J5***14**, *z2***15**, *z4***16**, *z5***10**, *Z1***21**, *Z4***46**, *Z5***58**, *s4***18**, *s6***22**, *S5***23**, and *r3***15**. Seta *r3* located on the margin of dorsal shield. All setae inserted on tubercles, but only the tubercles of setae *Z4* and *Z5* large and distinctly visible. Setae *s6* and *J5* slightly serrated; setae *Z4* and *Z5* strongly serrated and distinctly thick.

***Peritreme*** Extending to the level of *j1*.

***Venter*** (Fig. 4B). Sternal shield barely discernible; distance between *st1-st3***50**, *st1-st1***48**, *st2-st2***54**, and between *st3-st3***58**. Metasternal plates not visible. Genital shield smooth, with distance between *st5-st5***50**. Ventrianal shield longer than wide, **95** long and **81** wide at the level of *ZV2*; smooth anteriad *JV3*, reticulate elsewhere; with four pairs of preanal setae (*JV1*, *JV2*, *JV3*, and *ZV2*) and a pair of rounded pores *gv3*, posteriad to *JV2*; distance between *gv3-gv3***9**. Unsclerotised cuticle next to the ventrianal shield with two pairs of setae (*ZV1* and *JV5*). One pair of metapodal plate. All ventral setae smooth.

***Spermatheca*** (Fig. 4C). Calyx of the spermatheca **33** long with half next to vesicle tubular and the other half medially bulged, narrowing down at both extremes; atrium globular.

***Chelicera*** movable digit **20** long, apparently with one tooth in addition to the apical tooth; fixed digit **22** long, apparently with one tooth in addition to the apical tooth.

***Legs*** (Fig. 4D). In all legs, dorsal setae on genu, tibia and basitarsus inserted in distinct tubercles, while ventral setae arise directly from the cuticle. Chaetotaxy as follows: trochanter I 0 0/1 0/1 0, genu I 2 2/1 2/1 2, genu II 2 2/0 2/0 1, genu III 1 2/1 2/0 1, genu IV 1 2/1 2//0 1.

**Figure 5.**
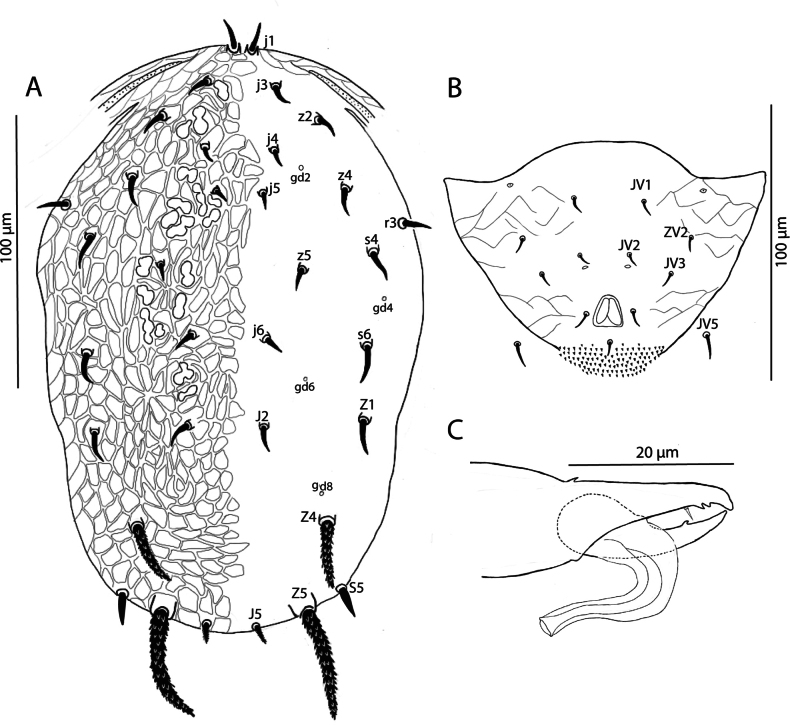
Galendromimus (Galendromimus) striatusornatus sp. nov., male. **A.** Dorsal idiosoma; **B.** Ventrianal shield; **C.** Spermatodactyl.

Male (*n* = 2). ***Dorsum of idiosoma*** (Fig. 5A). Setal pattern and dorsal shield ornamentation as in the female, except for the absence of the characteristic ornamentation between setae *Z4* and *Z5* observed in the female, visible pore *gd8*, and pore g*d9* not observed; 221–225 long and 128–146 wide at *s4* level; Four pairs of pores (*gd2*, *gd4*, *gd6* and *gd8*) visible. Lengths of setae: *j1* 11–13, *j3* 10, *j4* 8–10, *j5* 8, *j6* 9–11, *J2* 12, *J5* 9–11, *z2* 11–13, *z4* 12–14, *z5* 9–11, *Z1* 13–15, *Z4* 27, *Z5* 39–41, *s4* 11, *s6* 14–16, *S5* 11–13, *r3* 11–13. Seta *r3* located at the margin of the dorsal shield. All setae inserted in tubercles, but only the tubercles of setae *Z4* and *Z5* are large and distinctly visible. Seta *J5* slightly serrated, setae *Z4* and *Z5* strongly serrated and distinctly thick.

***Peritreme*** extending to level of *j3*.

***Venter*** (Fig. 5B). All ventral setae smooth. sternogenital shield smooth; ventrianal shield subtriangular with light lateral reticulation and with one pair of lyrifissures next to the anterior margin; 80–86 long and 121–123 wide at level of anterior corners, with four pairs of preanal setae (*JV1*, *JV2*, *JV3*, and *ZV2*). One distinct pair of pores (*gv3*) posterior to *JV2*.

***Chelicera*** fixed digit 18–20 long with two teeth between pilus dentilis and distal tooth, movable digit 16–18 long with one subapical tooth in addition to apical tooth.

***Spermatodactyl*** (Fig. 5C). c-shaped, with distal end facing back- and downward, shaft 8–10, foot 6–8.

***Legs*** as in females.

#### Differential diagnosis.

**Figure 6.**
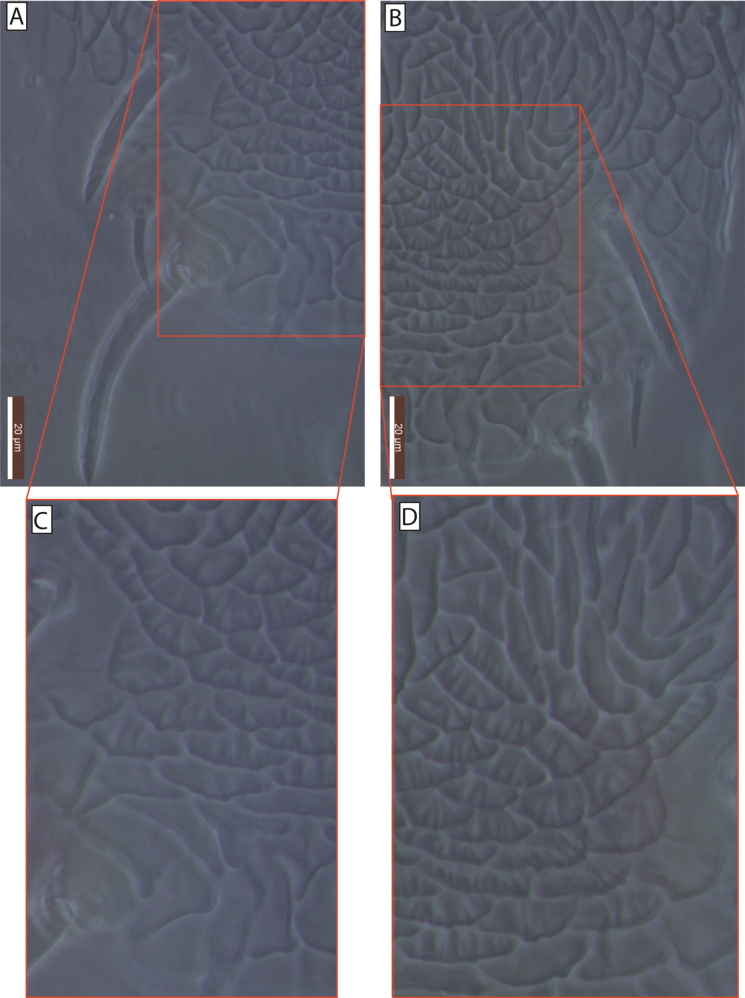
Galendromimus (Galendromimus) striatusornatus sp. nov. **A–D.** Ornamentation pattern in the central region of the female dorsal shield, between setae *Z4* and *Z5*.

Galendromimus (Galendromimus) striatusornatus sp. nov. is assigned to the *alveolaris* species group. Females of the new species differ from those of other species by having many striae present in the posterior region of the dorsal shield, between setae *Z4* and *Z5* (Fig. 6), which were not mentioned or illustrated in the description of any another species of this group. Females of the species in this group differ from the new species as subsequently mentioned. Galendromimus (G.) paulista, seta *J2* absent (present in the new species). Galendromimus (G.) alveolaris, peritreme extending to the level of seta *s4*; spermathecal calyx bulbous near atrium; seta *S5* 13 µm long and acicular. Galendromimus (G.) multipoculi with calyx tubular; ventrianal shield narrower than maximum width of genital shield; setae *z4*, *Z1*, *s4*, and *s6* ~ 2.1, 1.8, 1.7, and 1.5× longer than in the new species. Galendromimus (G.) primulaporis sp. nov., with the peritreme extending only to the level of seta *z2*; shorter setae *Z4* (25 µm) and *S5* (14 µm). Finally, G. (G.) tunapunensis differs by having serrated setae *z2*, *z4*, *Z1*, and *s4*; notably longer setae *Z1*, *Z4*, *s4*, and *s6*—being 3, 1.5, 2, and 2× longer, than in the new species.

#### Etymology.

The specific name *striatusornatus* is derived from the Latin *striatus* (striated, grooved) and *ornatus* (ornamented, decorated), in reference to the ornamentation of the dorsal shield between setae *Z4* and *Z5*, in which each reticle includes a series of parallel ridges perpendicular to the longest margin (Fig. 6).

##### ﻿Worldwide geographic distribution for Galendromimini species

Species of Galendromimini have been recorded from 19 countries across the Americas, Africa, and Asia, totalling 15 species distributed among four genera (Demite et al. 2025). South America harbours the highest species richness, with Brazil standing out as the most diverse country, harbouring nine of the 15 known species of the tribe. In Central America and the Caribbean, records include Costa Rica, Cuba, Puerto Rico, Jamaica, Trinidad, Martinique, and Marie Galante. Among these, Costa Rica, Jamaica, and Trinidad are the only countries in which more than one species have been reported. In North America, only Mexico and the United States of America (Florida) have confirmed records, each with one species. Outside the Americas, Galendromimini species have been reported in a few countries across Africa and Asia.

Understanding the geographic distribution of species within the tribe Galendromimini is essential to uncover their evolutionary history, ecological roles, and potential conservation needs. The results show that G. (G.) alveolaris is the most widely distributed species, recorded in nine countries across the Americas. In contrast, the majority of species, including *Breviseius sennae*, *Cydnoseis muntius*, *Cydnoseis vitis*, Galendromimus (G.) kyholithus, Galendromimus (G.) multipoculi, G. (G.) paulista, G. (G.) roraimensis, G. (G.) sanctus and G. (G.) tunapunensis, as well as the two new species described herein (Table 1) exhibit highly restricted ranges. This pattern, which accounts for approximately 70% of the species diversity in the tribe, suggests a strong tendency toward endemism within Galendromimini. The remaining 30% of species, including G. (G.) alveolaris, show broader distributions and may reflect greater ecological plasticity or wider dispersal abilities.

**Table 1.** Distribution of species from all genera of the tribe Galendromimini.

Species	ARG	BRA	COL	CRI	CUB	EGY	ISR	IND	JAM	MAR	MGA	MEX	MOR	OMA	PHI	PRI	TRI	USA	UAE	SAR
*Breviseius sennae*		X^31^																		
*Cydnoseius muntius*															X^32^					
*Cydnoseius negevi*						X^33,34^	X^35,36^	X^50^					X^37^	X^38^					X^42^	X^39, 40,41^
*Cydnoseius vitis*						X^43^														
G. (G.) alveolaris		X^1,2,3,4, 5,6,7,8, 9^	X^10^	X^11^	X^12,13, 14,15,16^				X^17^	X^18^		X^19,20^					X^21^	X^22^		
G. (G.) kynolithus		X^23,24^																		
G. (G.) multipoculi		X^5,6,25, 26,27^																		
G. (G.) paulista		X^28^																		
G. (G.) primulaporis sp. nov.		X^51^																		
G. (G.) roraimensis		X^29^																		
G. (G.) sanctus																	X^21^			
G. (G.) striatusornatus sp. nov.		X^51^																		
G. (G.) tunapunensis																	X^21^			
G. (N.) boriquensis									X^17^		X^18^					X^30^				
*Silvaseius barretoae*	X^44,48^	X^5, 6,44, 45,46, 47^		X^49^																

ARG: Argentina; BRA: Brazil; COL: Colombia; CRI: Costa Rica; CUB: Cuba; EGY: Egypt; ISR: Israel; IND: India; JAM: Jamaica; MAR: Martinique; MGA: Marie Galante; MEX: Mexico; MOR: Morocco; OMA: Oman; PHI: Philippines; PRI: Puerto Rico; TRI: Trinidad; UAE: United Arab Emirates; USA: United States of America; SAR: Saudi Arabia; 1. De Moraes et al. (1993); 2. Mendonça et al. (2019); 3. Ferla and de Moraes (2002); 4. Daud and Feres (2005); 5. Demite et al. (2011); 6. Demite et al. (2012); 7. Demite et al. (2013); 8. Feres and de Moraes (1998); 9. Zacarias and de Moraes (2001b); 10. de Moraes and Mesa (1988): 11. Chant and Baker (1965); 12. Hastie et al. (2010); 13. Ramirez and Ramos (1987); 14. Ramos et al. (2007); 15. Ramos and de Moraes (2007); 16. Ramos and Rodriguez (2004); 17. Denmark and Muma (1978); 18. De Moraes, Kreiter and Lofego (2000); 19. De Leon (1959); 20. Denmark and Evans (2011); 21. De Leon (1967); 22. De Leon (1957); 23. Silva et al. (2024); 24. Rossetti et al. (2025); 25. Demite et al. (2017); 26. Mendonça et al. (2019); 27. Zacarias et al. (2002); 28. Zacarias and de Moraes (2001a); 29. Demite et al. (2014a); 30. De Leon (1965); 31. de Moraes, Barbosa and Castro (2013); 32. Schicha and Corpus Raroz (1992); 33. Abo-Shnaf and de Moraes (2014); 34. Nasr et al. (2011); 35. Palevsky et al. (2009); 36. Swirski and Amitai (1961); 37. Zahidi et al. (2023); 38. Al-Shanfari et al. (2013); 39. Alatawi et al. (2017); 40. Alatawi et al. (2018); 41. Negm et al. (2012); 42. Negm (2014); 43. Basha et al. (2001); 44. Demite et al. (2008); 45. Demite et al. (2009); 46. Buosi et al. (2006); 47. Feres et al. (2007); 48. Guanilo et al. (2008); 49. Chant and Yoshida-Shaul (1991); 50. Jose et al. 2024; 51. This study.

It is important to recognise that observed patterns of species richness and distribution may be influenced by differences in research focus. In Africa, for example, studies on Phytoseiidae have focused on their role in agricultural systems, including the biological control of the cassava green mite (Mutisya et al. 2017), whereas in Brazil, a considerable number of surveys have been conducted in natural environments (Daud and Feres 2005; Castro and Moraes 2007; Mendonça et al. 2019; Silva et al. 2024). This contrast suggests that species of Galendromimini may preferentially inhabit natural habitats, which could explain the higher number of recorded species in Brazil. These findings emphasise the importance of continued biodiversity surveys and taxonomic revisions, particularly in underexplored habitats, to fully comprehend the diversity and distributional patterns of this tribe. Such knowledge is crucial for informing conservation strategies and understanding the ecological dynamics of these predatory mites.

### ﻿Key to species of Galendromimini of the world (updated from Silva et al. 2024)

**Table:** 

1	Setae *S2* and *S4* present	**2**
–	Setae *S2* and *S4* absent	**5**
2	Seta *Z1* absent. *Breviseius* Moraes, Barbosa & Castro	***B. sennae* Moraes, Barbosa & Castro**
–	Seta *Z1* present. *Cydnoseius* Muma	**3**
3	Leg IV without macrosetae	***C. muntius* Schicha & Corpuz-Raros**
–	Leg IV with macrosetae	**4**
4	Dorsal shield with scale-like reticules; sternal and ventrianal shields reticulate; genital shield reticulated laterally and smooth centrally	***C. negevi* (Swirski & Amitai)**
–	Anterior half of dorsal shield transversely striate and posterior half with transversely elongate reticules; ventral shields smooth	***C. vitis* Basha, Yousef, Ibrahim & Mostafa**
5	Seta *z3* absent; *Z1* absent and *R1* present. *Silvaseius* Chant & McMurtry	***S. barretoae* (Yoshida-Shaul & Chant)**
–	Seta *z3* present/absent; *Z1* present and *R1* absent. *Galendromimus* Muma	**6**
6	Setae *J2* and *JV3* absent; *JV4* and *ZV3* present; calyx of spermatheca cup-shaped; *JV5* stout and serrated. Galendromimus (Nothoseius) De Leon	***G.* (*Nothoseius*) *borinquensis* (De Leon)**
–	Seta *J2* present/absent, seta *JV3* present; *JV4* and *ZV3* absent; calyx of spermatheca variable; *JV5* setiform, smooth or serrated Galendromimus (Galendromimus) Chant & McMurtry	**7**
7	Seta *S5* absent	**8**
–	Seta *S5* present. *alveolaris* species group	**10**
8	Seta *z3* present. *roraimensis* species group	**G. (*G.*) *roraimensis* Demite & Lofego**
–	Seta *z3* absent. *sanctus* species group	**9**
9	Seta *Z1* not reaching the base of *Z4*	**G. (*G.*) *sanctus* De Leon**
–	Seta *Z1* reaching the base of *Z4*	**G. (*G.*) *kynolithus* Silva, Gondim Jr & Demite**
10	Seta *J2* absent; *JV5* serrated	**G. (*G.*) *paulista* Zacarias & Moraes**
–	Seta *J2* present; *JV5* smooth	**11**
11	Peritreme short, not reaching beyond *z2*	**12**
–	Peritreme extending to level of *j1*	**13**
12	Setae *Z4* and *Z5* with nearly equal lengths	**G. (*G.*) alveolaris (De Leon)**
–	Seta *Z4* is less than half the length of *Z5*	**G. (*G.*) primulaporis sp. nov.**
13	With many distinct “pits” on the central region of the dorsal shield; *r3* on unsclerotised cuticle	**G. (*G.*) multipoculi Zacarias, Moraes & McMurtry**
–	Without “pits” on the central region of the dorsal shield; *r3* on dorsal shield	**14**
14	Setae *z2*, *z4*, *s4*, and *Z1* serrated; seta *J5* smooth	**G. (*G.*) tunapunensis De Leon**
–	Setae *z2*, *z4*, *s4*, and *Z1* smooth; seta *J5* serrated	**G. (*G.*) striatusornatus sp. nov.**

## Supplementary Material

XML Treatment for Galendromimus

XML Treatment for Galendromimus (Galendromimus)

XML Treatment for Galendromimus (Galendromimus) primulaporis

XML Treatment for Galendromimus (Galendromimus) striatusornatus

## ﻿Additional information

### Use of AI

No use of AI was reported.

### Author ORCIDs

Amanda Conceição dos Santos https://orcid.org/0000-0001-6212-5739

Peterson Rodrigo Demite https://orcid.org/0000-0001-6376-5463

Felipe Micali Nuvoloni https://orcid.org/0000-0001-9642-557X

Antonio Carlos Lofego https://orcid.org/0000-0002-3495-6858

### Data availability

All of the data that support the findings of this study are available in the main text.
